# Extracellular Matrix Stiffness-Induced Mechanotransduction of Capillarized Liver Sinusoidal Endothelial Cells

**DOI:** 10.3390/ph17050644

**Published:** 2024-05-16

**Authors:** Qingjuan Wu, Quanmei Sun, Qiang Zhang, Ning Wang, Wenliang Lv, Dong Han

**Affiliations:** 1 Guang Anmen Hospital, China Academy of Chinese Medical Sciences, Beijing 100010, China; 18253199390@163.com (Q.W.); wangn2021@nanoctr.cn (N.W.); 2 National Center for Nanoscience and Technology, Beijing 100190, China; sunqm@nanoctr.cn; 3 Hebei Key Laboratory of Nano-Biotechnology, Yanshan University, Qinhuangdao 066104, China; zhangq@ysu.edu.cn

**Keywords:** liver sinusoidal endothelial cells, NO-dependent pathway, liver fibrosis, biomechanics

## Abstract

The mechanobiological response mechanism of the fenestrae of liver sinusoidal endothelial cells (LSECs) to the physical stiffness of the extracellular matrix (ECM) remains unclear. We investigated how the mechanical properties of their substrates affect the LSECs’ fenestrae by the nitric oxide (NO)-dependent pathway and how they relate to the progression of hepatic sinus capillarization during liver fibrosis. We detected different stiffnesses of ECM in the progress of liver fibrosis (LF) and developed polyacrylamide hydrogel (PAM) substrates to simulate them. Softer stiffness substrates contributed to LSECs maintaining fenestrae phenotype in vitro. The stiffness of liver fibrosis tissue could be reversed in vivo via treatment with anti-ECM deposition drugs. Similarly, the capillarization of LSECs could be reversed by decreasing the ECM stiffness. Our results also indicate that the NO-dependent pathway plays a key regulatory role in the capillarization of ECM-LSECs. Our study reveals ECM-induced mechanotransduction of capillarized LSECs through a NO-dependent pathway via a previously unrevealed mechanotransduction mechanism. The elucidation of this mechanism may offer precise biomechanics-specific intervention strategies targeting liver fibrosis progression.

## 1. Introduction

Liver fibrosis (LF) is a chronic disease characterized by the deposition of the extracellular matrix (ECM) [1]. It has been reported that, in 2020, chronic liver disease accounted for two million deaths and was responsible for 4% of all deaths; approximately 2 million people die every year due to varying degrees of chronic liver disease; the prevalence of advanced LF in the Chinese general population in 2023 reached 2.85% [2,3]. During the development of LF, the total protein content can increase by nearly 10-fold with abnormal deposition of ECM, resulting in liver stiffness increasing by ~6-fold compared to a normal liver [4,5]. The change in liver stiffness caused by ECM deposition is not only a result of the development of fibrosis but also the reason for further disease progression [6]. ECM is a crucial component of the microenvironment for liver sinusoidal endothelial cells (LSECs), so the phenotype of LSECs’ fenestrae and the balance between LSECs and other parenchymal liver cells are inevitably influenced by ECM biomechanical features in LF progression [7,8]. The accumulation of ECM restricts the blood flow of hepatic sinusoidal microvessels, causing stenosis and deformation of the hepatic sinusoid lumen, resulting in hypoxia and aggravated capillary vascularization. The crosstalk between ECM and LSECs could be explained by mechanotransduction, the mechanism by which force transmission between ECM and cells as well as between cells themselves is transformed into a series of intracellular signaling events [9].

The capillarization of LSECs, also known as liver sinusoid capillarization, is associated with the loss of fenestrae and the development of the basement membrane, which is an important pathological feature of LF [7,10]. The nitric oxide (NO)-dependent pathway plays a vital role in the development and regulation of fenestrae, and endothelial NO synthase (eNOS) is the main producer of NO in LSECs [11]. Most of the studies on the NO-dependent pathway in LSECs have focused on the biochemical mechanism [12]; however, the mechanical transduction from the ECM to the NO-dependent pathway in LSECs is still unclear. Recently, studies have shown that mechanical conduction during hemodynamic disturbances (such as low shear stress, blood flow perturbation, pathological stretch, and pressure increase) and ECM remodeling can regulate eNOS expression and NO synthesis in LSECs [13,14]. 

However, the questions of whether the physical mechanics of the ECM microenvironment also regulate the fenestrae of LSECs and what mechanism converts this biomechanical force into a mechanobiological response are still unanswered. Therefore, we explored the transmission of mechanical signals between ECM biomechanical features and LSECs, to understand if ECM biomechanical features may be targeted for intervention against the capillarization of LF.

## 2. Results

### 2.1. Changes in Fenestrae Phenotype Induced by Varying Substrate Stiffness

**Figure 1:**
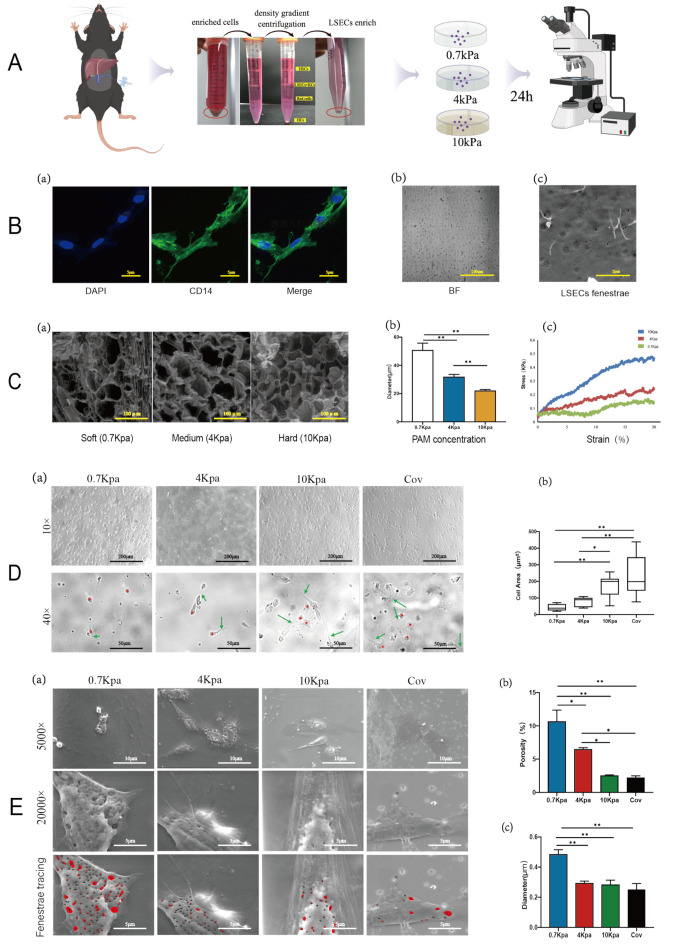
Different stiffness induces LSEC fenestrae phenotype change. (**A**). Flow chart of the experiment, and the primary LSECs are isolated which picked in red circle. (**B**). Identification and characterization of isolated LSECs. Fluorescence images of CD14 in LSECs, scale bar = 5 μm (a). Morphology of pure endothelial cells, typical cobblestone arrangement, scale bar = 200 μm (b). ESEM picture presented the sieve plates composed of clustered fenestrations, scale bar = 2 μm (c). (**C**). Characterization of PAM with different stiffnesses. ESEM pictures show the cross-sections of soft (0.7 kPa), medium (4 kPa), and hard (10 kPa) hydrogels with different stiffnesses. Scale bar = 100 μm (a). Pore diameters of soft, medium, and hard PAM hydrogel matrices after vacuum freeze-drying, *p* (0.7 kPa vs. 4 kPa) < 0.0001, *p* (0.7 kPa vs. 10 kPa) < 0.0001, *p* (4 kPa vs. 10 kPa) = 0.0004 (b). The tensile curves show an obvious gradient trend of hydrogels with varying stiffness (0.7 kPa, 4 kPa, and 10 kPa) (c). (**D**) Primary mice LSECs were extracted and cultured on different stiffness PAMs, and observed via environmental scanning electron microscopy. Time-lapse images of mice LSECs on substrates with different stiffnesses; magnifications are 10× and 40×, respectively. Asterisks for stalk lamellipodia; arrows for filopodia (a). Cell spreading area (including lamellipodia and filopodia) on different stiffnesses, *p* (0.7 kPa vs. 10 kPa) = 0.0001, *p* (4 kPa vs. 10 kPa) = 0.0281, *p* (4 kPa vs. Cov) = 0.0001, *p* (0.7 kPa vs. Cov) < 0.0001 (b). (**E**). ESEM pictures show the details of LSEC fenestrae which marked by red circle; magnifications are 5000× and 20,000×, respectively (a). Porosities of LSECs are 10.63%, 6.47%, 2.51%, 2.19% on stiffnesses of 0.7 kPa, 4 kPa, 10 kPa, and coverslip, respectively, *p* (0.7 kPa vs. 4 kPa) = 0.0147, *p* (0.7 kPa vs. 10 kPa) < 0.0001, *p* (0.7 kPa vs. Cov) < 0.0001, *p* (4 kPa vs. 10 kPa) = 0.0212, *p* (4 kPa vs. Cov) = 0.0118 (b); diameters are 0.484 ± 0.19 μm for 0.7 kPa, 0.293 ± 0.07 μm for 4 kPa, 0.283 ± 0.14 μm for 10 kPa, and 0.25 ± 0.16 μm for coverslip, *p* (0.7 kPa vs. 4 kPa) < 0.0001, *p* (0.7 kPa vs. 10 kPa) < 0.0001, *p* (0.7 kPa vs. Cov) < 0.0001 (c). ∆*p* values were determined via one-way ANOVA; * indicates a significant difference at *p* < 0.05; ** indicates a very significant difference at *p* < 0.01. All data are presented as mean ± s.e.m.

Primary LSECs were cultured on hydrogels and scanned via electron microscopy; phenotypic biomarker CD14 [15] and typical sieve structures were observed (Figure 1A,B). PAM (polyacrylamide hydrogels) is commonly used to make hydrogels as it is non-toxic and stiffness adjustable [16,17,18,19]. Therefore, we used PAM to simulate a healthy liver (0.7 kPa), liver fibrosis progression (4 kPa), and late liver fibrosis/cirrhosis stage (10 kPa) tissues, while we set coverslips (stiffness up to 30 GPa) as a control group. We characterized the stiffness, porosity, and tensile curves of these hydrogel substrates, which show a gradient trend (Figure 1C(a–c)). Freshly isolated primary mouse LSECs can maintain normal fenestration phenotype for up to 72 h in in vitro culture [20,21,22]. Therefore, we examined them for lamellipodia and filopodia via fiberoptic microscopy after the isolated LSECs were cultured on different stiffness substrates for 24 h (Figure 1D(a)). The lamellipodia, filopodia, and intercellular connections decreased, implying decapillarization, with decreasing substrate stiffness (Figure 1D(a,b)). We used ESEM (Environmental Scanning Electron Microscope) to observe the changes in fenestrae morphology to further test the effect of substrate stiffness (Figure 1E). As matrix stiffness increased, cell spreading increased with higher amounts of lamellipodia and filopodia (Figure 1E(a)). There was a sharp decrease in fenestrae from the softer to harder substrates, and the porosities of LSECs were 10.63%, 6.47%, 2.51%, and 2.19% on substrate stiffnesses of 0.7, 4, 10 kPa, and coverslips, respectively (Figure 1E(b)). Moreover, an increase in fenestrae diameters was associated with a decrease in matrix rigidity (0.484 ± 0.19, 0.293 ± 0.07, 0.283 ± 0.14, and 0.25 ± 0.16 μm for 0.7, 4, 10 kPa, and coverslips, respectively) (Figure 1E(c)). These results indicated that the fenestrae LSECs are regulated by substrate stiffness.

### 2.2. Stiffness Modulates Capillarization of LSECs by Regulating the Cytoskeleton 

**Figure 2:**
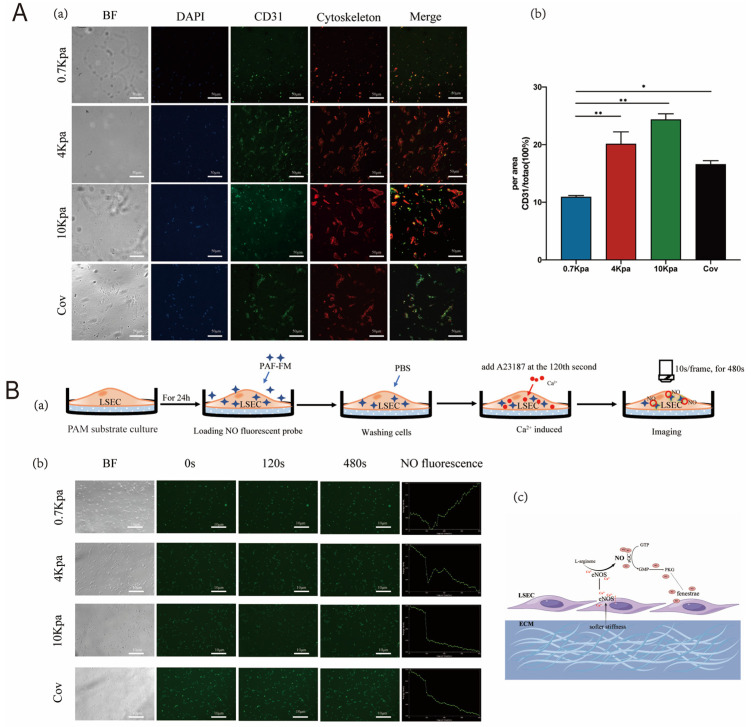
Stiffness modulates LSEC capillarization by regulating cytoskeleton and NO-dependent pathway. (**A**). Immunostaining of LSECs afer 24 h of culture on PAM hydrogels with different stiffnesses. Cells labeled for nucleus (blue), cytoskeleton (red), and CD31 (green). Scale bar = 50 μm; data are presented as mean ± s.e.m. (a). Quantification of CD31 after cells cultured for 24 h, *p* (0.7 kPa vs. 4 kPa) < 0.0001, *p* (0.7 kPa vs. 10 kPa) < 0.0001, *p* (0.7 kPa vs. Cov) = 0.0117 (b). (**B**) NO release on different stiffnesses induced by A23187. Flow diagram, NO fluorescence signal intensity is recorded in real time, A231187 was added at the 120th second, and real-time monitoring is continuous for 480 min. Fluorescence pictures were collected 10 s/frame and saved for relative enhancement analysis (a). The relative enhancement of NO fluorescence signal on soft (0.7 Kpa), medium, and high stiffness, and coverslip are 1.65%, −3.3%, −4.05%, and −4.83%, respectively (b). Mechanism diagram of NO release induced by Ca^2+^ ions (c). ∆*p* values were determined via one-way ANOVA; * indicates *p* < 0.05, ** indicates *p* < 0.01.

To explore the effect of substrate stiffness on stress fibers, isolated LSECs were cultured on different stiffness PAMs for up to 24 h (Figure 2A). LSECs exhibited a complete re-organization of their actin cytoskeleton with an increase in stress fiber formation with the increase in substrate stiffness (Figure 2A(a)). The expression levels of CD31 were elevated, indicating the capillarization of liver sinusoids [23]. Fluorescence images were used to analyze the density of CD31 on the cell surface. CD31 expression was higher on the harder PAM substrate than 0.7 kPa substrate (Figure 2A(b)) demonstrating increased capillarization associated with increased stiffness (mean fluorescence intensities were 10.91 ± 0.82, 20.12 ± 6.66, 24.35 ± 3.2, and 16.58 ± 2.03 for 0.7, 4, 10 kPa, and coverslip, respectively). Unexpectedly, the CD31 intensity did not show a continuous trend of increasing with substrate stiffness, and the intensity of CD31 on cells on the hardest substrate (glass coverslip) was lower than those on 4 and 10 kPa hydrogel substrate, although it was higher than those on the 0.7 kPa substrate. This interesting phenomenon intimated that the isolated LSECs underwent a spontaneous dedifferentiation process on common basal dishes in vitro (whose stiffness is similar to glass coverslips and far beyond the stiffness range of liver tissue in vivo). Thus, they are not a valid biological representation of real LSECs, which suggests the importance of choosing reasonable substrate stiffness parameters in in vitro experiments.

### 2.3. Stiffness Regulated Fenestrae of LSECs through the NO-Dependent Pathway

To analyze whether the regulation of LESC fenestrae on different substrate stiffnesses was linked to the NO-dependent pathway, we loaded DAF-FM DA (a NO fluorescent probe) onto LESCs before seeding on different stiffness substrates, and we used the Ca^2+^ carrier A23187 to activate endothelial nitric oxide synthase (eNOS) after 24 h. We recorded the real-time fluorescence changes brought by NO release at 10 s/frame (Figure 2B(a)). The NO signal intensity (SI) in all groups showed a declining trend before intervention (0–120 s), which was the normal phenomenon for SI attenuation. However, when we added the eNOS activator A23187 at 120 s, only the cells on the softest substrate (0.7 kPa) exhibited an increase in SI, while the three other groups (4 kPa, 10 kPa, and coverslip) still showed a continuously declining trend. We calculated the relative enhancement (RE) of NO SI to be 1.65%, −3.3%, −4.05%, and −4.83% on 0.7, 4, 10 kPa, and coverslip, respectively (Figure 2B(b)). These results suggested that eNOS was the key enzyme to regulate NO synthesis and release in LSECs, and the softer substrate (0.7 kPa) contributed to LSECs maintaining eNOS activity (Figure 2B(c)). In contrast, 4 kPa and higher stiffness substrates did not enable LSECs to maintain eNOS activity or even disrupt it, so they could not be induced to release NO by Ca^2+^ influx.

### 2.4. Silybin Treatment In Vivo Contributed to ECM Composition Changes 

**Figure 3:**
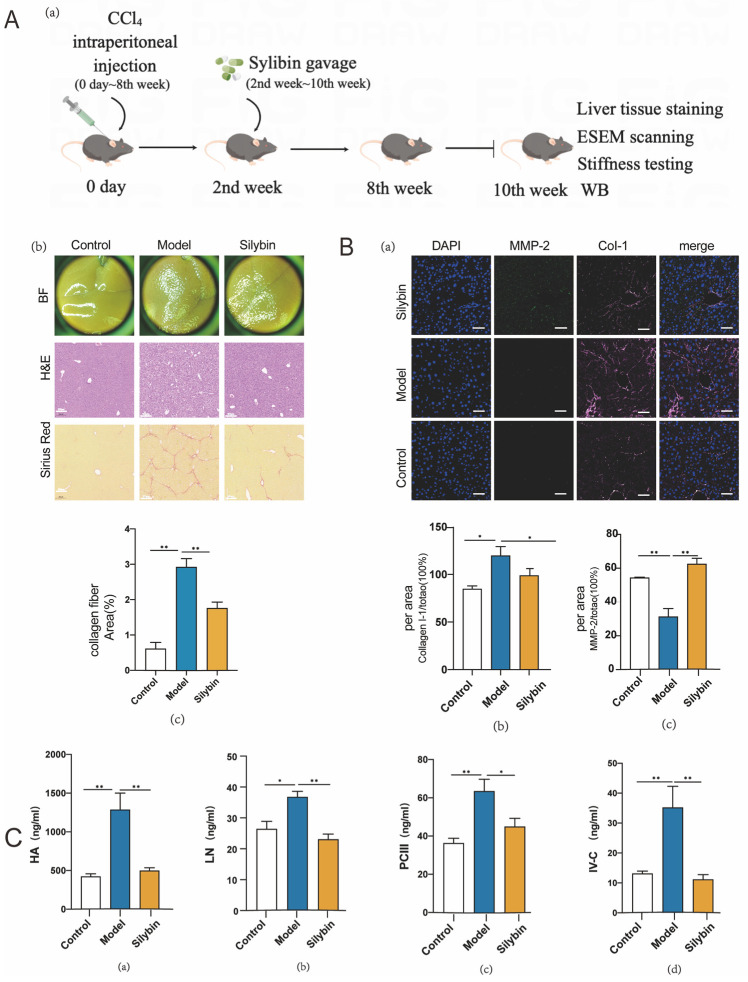
Extracellular matrix composition features of silybin-treated mice fibrosis liver. (**A**) Daily silybin treatment was performed 8 weeks for CCl_4_-induced liver fibrosis mice. Flow diagram (a). Brightfield pictures show the liver surface particle sensation in model and silybin groups; HE and Sirius red staining pictures show the deposition of collagen fibers (scale bar = 200 μm, Col-I is stained red in Sirius red staining) (b). Statistical analysis of the collagen fiber area on Sirius red staining, *p* (control vs. model) < 0.0001, *p* (model vs. silybin) = 0.0017 (c). (**B**) Nucleus (blue), MMP-2 (green) and Col-I (pink) were stained with immunostaining, in all panels, scale bar = 50 μm (a). Statistical analysis of the expression of Col-I, *p* (control vs. model) = 0.0254, *p* (model vs. silybin) = 0.0495 (b) and MMP-2, *p* (control vs. model) = 0.0087, *p* (model vs. silybin) = 0.0003 (c), and between different groups. (**C**) Serum concentrations of HA (*p* (control vs. model) < 0.0001, *p* (model vs. silybin) = 0.0003), LN (*p* (control vs. model) = 0.0149, *p* (model vs. silybin) = 0.0008), PCIII (*p* (control vs. model) = 0.0005, *p* (model vs. silybin) = 0.0354), IV-C (*p* (control vs. model) = 0.0024, *p* (model vs. silybin) = 0.0015) in different animal groups (a–d). ∆*p* values were determined via one-way ANOVA; * indicates *p* < 0.05, ** indicates *p* < 0.01. N = 6; all data are presented as mean ± s.e.m.

When liver fibrosis occurs, ECM mainly includes interstitial matrix (type I (Col-I) and IV (Col-IV) collagen fibers [24,25], matrix metalloproteinases (MMPs) [26]) and the basement membrane (a cuff-like structure generated on capillarization of LSECs). We analyzed the collagen fiber deposition in the healthy (control), CCl_4_-induced liver fibrosis (model), and silybin-treated liver fibrosis (silybin) groups via HE staining and Sirius red staining (Figure 3A(a)). Brightfield images showed that the silybin-treated group’s liver surface roughness was improved compared to the model group, although it was not as smooth as the control group. Correspondingly, HE staining images in the treatment group also indicated that the inflammation of the hepatocytes and damage to the hepatic lobules were relieved compared to the model group, and Sirius red staining images showed that the silybin-treated group could significantly reduce the collagen fiber deposition in fibrosis liver (Figure 3A(b)). Statistical analysis of the collagen fiber deposition area showed that the silybin-treated group showed a significantly lower deposition area than the model group (Figure 3A(c)). When we labeled Col-I and MMP-2 in liver tissue via immunofluorescence (IF) straining, statistical analysis revealed that the expression of Col-I decreased while the MMP-2 expression increased significantly in the silybin-treated group compared to the model group (Figure 3B(a–c)). The basement membrane is composed of type III procollagen (procollagen III, PCIII), type IV collagen (collagen IV, IV-C), laminin (laminin, LN), and hyaluronan (hyaluronan, HA). To assess the effectiveness of silybin on these ECM components, we tested these four indexes in mice serum. Results showed that silybin could significantly reduce the levels of all four indicators (Figure 3C(a–d)). These findings suggest that silybin could alleviate liver fibrosis to some extent, which leads to a downstream effect on the ECM components (interstitial matrix and basement membrane).

### 2.5. Biomechanical Alteration after Silybin-Treatment of Mice with Liver Fibrosis 

**Figure 4:**
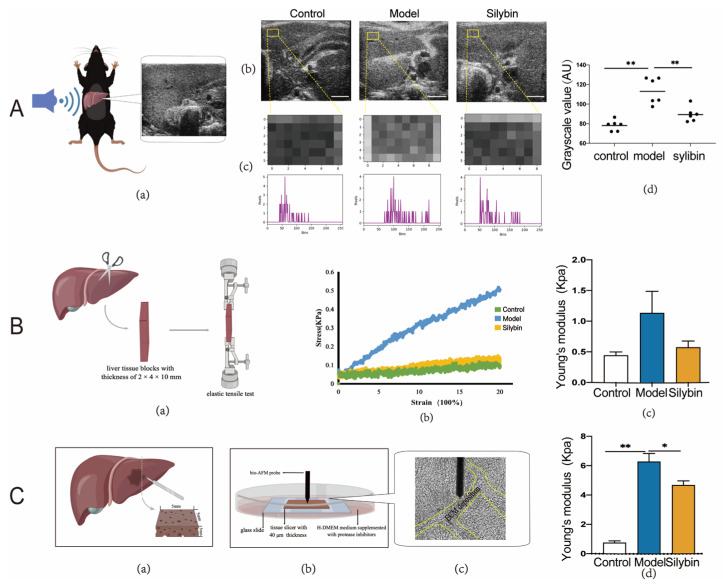
Biomechanical measurement of silybin-treated fibrosis mice liver. (**A**). Mice liver ultrasonography detection in vivo. Flow diagram (a). The grayscale images of mice liver, control (healthy mice), model (CCl_4_-induced fibrosis mice), and silybin (silybin treatment) groups, scale bars = 2 mm (b). The grayscale histogram is to count the grayscale value of each pixel point and draw it as a histogram, the horizontal axis is the grayscale value (0, 255), and the vertical axis is the number of pixels corresponding to the grayscale value. We draw the histogram of grayscale images by the OpenCV cv2.calcHist function in the Python module (c). Statistical analysis of the difference of gray value between each grayscale images, *p* (control vs. model) < 0.0001, *p* (model vs. silybin = 0.0013 (d). (**B**) Elasticity detection of liver tissues via elastic tensile tester. Flow diagram (a). The elastic tensile curve of liver tissue and the statistical analysis of Young’s modulus (b,c). (**C**). AFM analyzes the nanomechanical profiles of ex vivo liver tissues under physiological buffer conditions, flow diagram (a–c). The Young’s modulus values are 0.74 ± 1.25 kPa, 6.27 ± 4.42 kPa, 4.67 ± 2.84 kPa in control group, model group, and silybin-treated groups, respectively, *p* (control vs. model) < 0.0001, *p* (model vs. silybin = 0.0234 (d). ∆*p* values were determined via one-way ANOVA; * indicates *p* < 0.05, ** indicates *p* < 0.01. N = 6; all data are presented as mean ± s.e.m.

To macroscopically evaluate the biomechanical liver profiles in different groups, we performed liver ultrasonography in vivo (Figure 4A(a)). The liver area on the body surface was fully exposed, and we performed ultrasound scanning using the small probe at a constant probing depth, recording grayscale images of the livers. The images of normal livers (with soft liver stiffness) showed a higher number of darker pixels, while a higher number of brighter pixels on fibrosis liver images suggested a higher liver stiffness (Figure 4A(b)). We chose three regions of interest (ROIs) randomly on the left liver lobe to calculate mean grayscale values to evaluate the difference in liver stiffness. Statistical analysis showed that silybin treatment significantly reduced grayscale values in mice with fibrosis (Figure 4A(c,d)), which suggested a restorative effect of silybin on fibrosis liver stiffness. As the Young’s modulus is used to describe the mechanical characteristics of materials, we used it to characterize the stiffness of isolated mouse liver tissue. We detected the elasticity of liver tissues using an elastic tensile tester and calculated Young’s modulus according to the measured tensile curve (Figure 4B(a,b)). Silybin treatment could significantly reduce Young’s modulus of the liver tissues (Figure 4B(c)). To further elucidate the nanomechanical profiles in silybin-treated biopsies, we performed bio-AFM analyses of liver tissues ex vivo under physiological buffer conditions. We examined ECM deposition at multiple locations per sample. The experimental approach is described in Figure 4C(a–c). Calculating Young’s modulus of liver stiffness values from control mice revealed a healthy liver stiffness distribution of 0.74 ± 1.25 kPa, while the fibrosis liver tissue with its characteristic high ECM deposition showed an increased stiffness of 6.27 ± 4.42 kPa. In comparison, a representative silybin-treated liver tissue exhibits softer features at 4.67 ± 2.84 kPa (Figure 4C(d)). All the results in our in vivo testing and in vitro macroscopic as well as microscopic mechanical characterization indicated that the stiffness of liver tissue had been greatly restored after treatment. 

### 2.6. Improved Fenestration Phenotype after Silybin Treatment of Liver Fibrosis 

**Figure 5:**
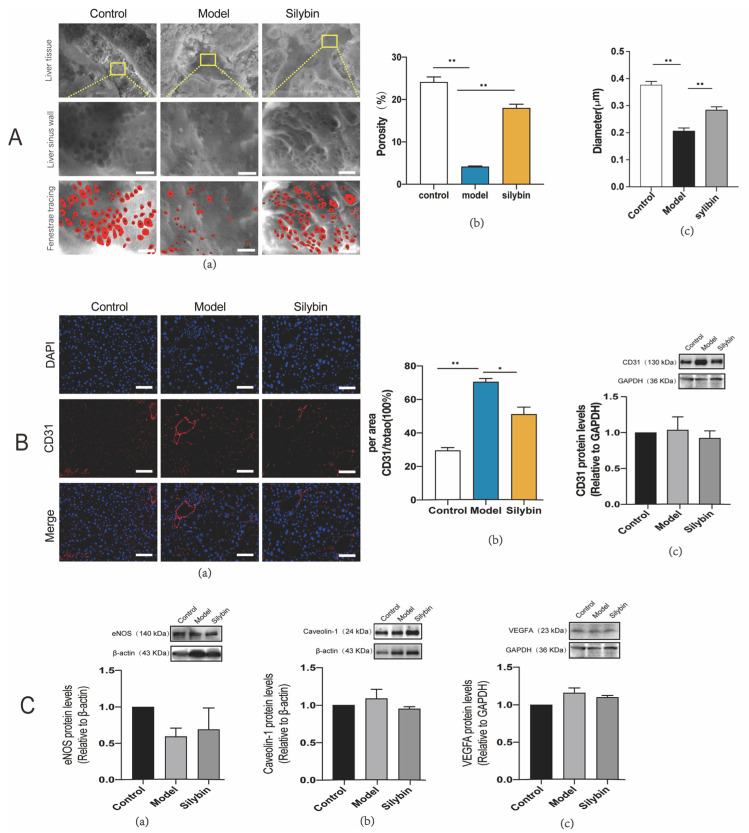
The softer silybin-treated liver tissue induced mechanotransduction of anti-capillarization via NO-dependent pathway. (**A**) ESEM pictures of the fenestration morphology on the surface of the hepatic sinusoids. Fenestrae are labeled in red circle by manual tracing, scale bar = 1 μm (a). Statistical analysis of the difference in fenestrae number, *p* (control vs. model) < 0.0001, *p* (model vs. silybin < 0.0001), (b) and diameter, *p* (control vs. model) < 0.0001, *p* (model vs. silybin = 0.0004) (c). (**B**) Immunostaining of liver tissue with nucleus (blue) and CD31 (red), and western blot of CD31 expression in liver tissue. In all immunostaining pictures, scale bar = 50 μm (a). Statistical analysis of the expression difference of CD31 in immunostaining pictures, *p* (control vs. model) < 0.0001, *p* (model vs. silybin = 0.0266 (b) and western blotting semi-quantitative assay for CD31 protein expression in liver tissue (c). (**C**). Western blotting semi-quantitative assay for the NO-dependent pathway proteins, eNOS, VEGFA, and caveolin-1. The results were confirmed by at least three independent experiments. Representative results of at least three independent experiments are shown (a–c). ∆*p* values are determined via one-way ANOVA * indicates *p* < 0.05, ** indicates *p* < 0.01. N = 6; all data are presented as mean ± s.e.m.

To further verify whether the ECM stiffness in vivo can induce capillarization of LSECs, we fixed mice liver via in situ perfusion and observed the morphology of LSEC fenestrae on the surface of hepatic sinusoids via ESEM (Figure 5A). As expected, the softer liver tissue in silybin-treated mice exhibited a more native fenestrae phenotype (Figure 5A(a)), and the porosities were 24.11%, 4.13%, and 17.96% for the control, model, and silybin-treated groups, respectively (Figure 5A(b)); and the mean diameters were 0.377 ± 0.10, 0.207 ± 0.06, 0.284 ± 0.11 μm for the control, model, and silybin-treated groups, respectively (Figure 5A(c)). The results of immunofluorescence labeling and western blot on CD31 in liver tissue sections were also consistent with the results of the ESEM (Figure 5B(a)), and silybin treatment significantly reduced CD31 expression in mice with fibrosis (Figure 5B(b,c)). These research results indicate that the ECM biomechanical changes caused by silybin treatment triggered the mechanotransduction phenomenon, thereby inducing a medicinal effect against hepatic sinusoidal capillarization. However, we cannot rule out a direct pharmacological effect of silybin in the reversal of capillarization, which needs to be verified in future experiments.

### 2.7. Silybin-Induced Mechanotransduction of Anti-Capillarization by the NO-Dependent Pathway

Besides eNOS involved in the NO-dependent pathway, the maintenance of LSEC fenestrae phenotype requires vascular endothelial growth factor A (VEGFA) [27,28], which is secreted by either hepatocytes or hematopoietic stem cells and can stimulate NO release from eNOS in LSECs [22,29,30,31]. In contrast, caveolin, the principal structural protein in caveolae, interacts with eNOS, which can modulate cellular regulation of NO synthesis and regulate LSEC fenestration [32,33]. To examine whether the nanomechanical changes associated with the fenestrae are related to NO-dependent regulatory proteins, we extracted proteins from liver tissue and performed western blot detection for a semi-quantitative analysis of the NO-dependent pathway regulatory proteins. To determine the expression pattern of eNOS, caveolae-1, and VEGFA when regulating hepatic sinusoid capillarization, we assessed the levels of these proteins in fibrosis model mice. eNOS was significantly down-regulated in fibrosis (harder liver stiffness); however, the expression of eNOS was increased after silybin treatment (softer liver stiffness) (Figure 5C(a)), which is similar to the results obtained in the in vitro real-time fluorescence record. Unlike the down-regulation of eNOS, VEGFA and caveolin-1 were both up-regulated in the model group, and the treatment with silybin decreased their levels (Figure 5C(b,c)). These results suggested that eNOS, VEGFA, and caveolin-1 play a key role in the NO-dependent pathway and were involved in the regulation of hepatic sinusoidal capillarization.

## 3. Discussion

Existing anti-sinusoidal capillarization chemotherapies focus on drugs’ biochemical effects. However, biomechanical forces also play an important role in the development and reversal of liver fibrosis [33,34,35,36]. ECM deposition in LF is often accompanied by changes in mechanical properties of the microenvironment; therefore, focusing on the effect of ECM mechanical force on LSEC capillarization can provide a potential intervention target in liver fibrosis treatment. Although in vitro studies have revealed a regulatory effect of ECM physical stiffness on LSEC angiogenesis and fenestrae [37,38], there is a lack of validation in vivo studies. We designed this study to explore the mechanism of ECM stiffness in regulating LSEC fenestrae both in vitro and in vivo. 

In the in vitro section of the study, we had to address substrate stiffness parameters and substrate simulation materials. First, we chose a linear elastic polyacrylamide hydrogel as the substrate material to mimic ECM stiffness, with good biocompatibility and widely tunable properties [16,39] to reproduce the mechanical environment experienced by cells in vivo [40]. Then, we obtained the reliable stiffness requirements by measuring liver tissue from mice. In previous reports about liver ECM stiffness, the stiffness gradient was set from 90 Pa to 36 kPa; however, the previous mice liver stiffness measured via the magnetic resonance method was 0.2–0.5 kPa, rat liver tissue measured via a Rheometrics RFS-3 controlled strain rheometer was 0.3–13 kPa, and human liver stiffness measured via the non-invasive liver elasticity test varied significantly between 2.4 and 75.4 kPa. This variation is due to differences in animal species (including rats, mice, ducks, etc.) and measurement methods; therefore, in order to set a substrate stiffness parameter in vitro that is closer to the biological liver stiffness, we measured the Young’s modulus of liver tissue via bio-AFM. Results showed that the stiffness of mice liver tissue was between 0.74 ± 1.25 kPa and 6.27 ± 4.42 kPa, which provided a reliable parameter for setting the hardness of the substrate.

Since in vitro experiments demonstrated the effect of substrate stiffness on the LSEC fenestrae, the in vivo crosstalk between ECM biomechanical features and LSEC capillarization needed to be proved. In the in vivo experiments, we chose silybin as the treatment drug for anti-liver fibrosis. We combined liver ultrasonography, elastic tensile tester, and bio-AFM to measure the biomechanical signatures after silybin treatment. Silybin treatment decreased ECM stiffness of fibrosis liver in all our measurements. In subsequent experiment, we further observed the reversion of hepatic sinusoidal capillarization in liver tissue with lowered stiffness. These findings suggested that silybin decreases the stiffness of the fibrotic liver by adjusting the biomechanical properties of the ECM, and the liver sinusoid capillarization was reversed with decreasing stiffness. These results indicate that biomechanics could be a potential new intervention in disease treatment. 

**Figure 6:**
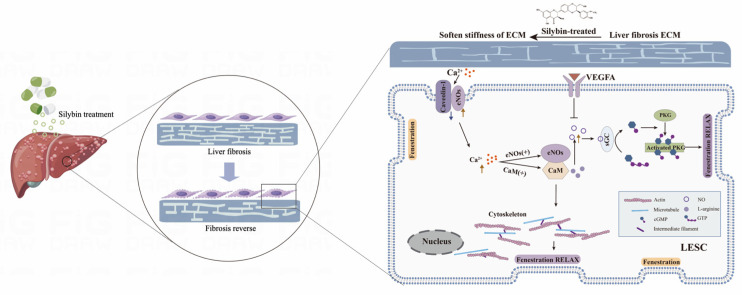
NO-dependent pathway response of LSECs to ECM stiffness. Endothelial phenotype is constantly modulated by both biomechanical and biochemical stimuli. Ca^2+^ ion channel and endothelial cytoskeleton are the mechanosensors interpreting ECM stiffness in LSECs. Firstly, silybin reduces tissue stiffness in liver fibrosis by reducing ECM deposition. Next, decreasing ECM stiffness up-regulates an influx in Ca^2+^, resulting in activation of eNOS; at the same time, eNOS is also activated by stimuli such as VEGF and sinusoidal blood flow shear stress; and then the NO produced by activated eNOS. Then, the NO pathway working through soluble guanylate cyclase, cGMP, and protein kinase G results in maintaining the normal fenestration topography of liver sinusoids and controls intrahepatic sinusoidal vascular tone and blood flow. On the other hand, influx Ca^2+^ induced the movement of the endothelial cytoskeleton and fenestrae regulated protein caveolin-1, further controlling the fenestrae phenotype.

We further aimed to reveal the key role of the NO-dependent pathway in the mechanotransduction mechanism between ECM and LSECs. We observed the eNOS activator (A23187)-induced NO release by the activation of eNOS on the softer substrate, which illustrated that eNOS plays the key role in the NO-dependent pathway involved in ECM-LSEC mechanotransduction. Further, NO, in turn, acts through soluble guanylatecyclase (sGC), conversion of GTP to cGMP, and stimulation of protein kinase G (PKG), which help to maintain the LSEC fenestrae [19,26,27,28]. Moreover, A23187 activated CaM, subsequently regulating the cytoskeletal topography, and ultimately contributed to the phenotypic changes in LSEC fenestrae. In contrast, along with the eNOS activation, the activities of caveolin-1 and VEGFA were antagonized in vivo, which involved the negative regulation of NO release in LSECs (Figure 6).

Overall, our studies indicate that softer stiffness substrates contribute to LSECs maintaining fenestrae phenotypic, and LSEC capillarization could be reversed by a decrease in ECM stiffness. We observed the importance of the NO-dependent pathway in the mechanotransduction crosstalk between ECM stiffness and LSEC capillarization, which indicated that biomechanics could be a potential intervention in the treatment of liver fibrosis.

However, the present study is still quite preliminary, and we cannot rule out the direct anti-capillarization pharmacological effect of the anti-ECM deposition drug silybin in this experiment. As a natural liver-protection drug, silybin is the major active component present in 60–70% of silymarin extract, which shows good safety and therapeutic effect on liver fibrosis and liver cancer by reducing liver transaminase, promoting liver cell regeneration, etc. At present, most studies focus on the pharmacological effects of silybin and are confined to the biochemical mechanism at the cellular level; there are still few studies on its liver microenvironment regulation and ECM component regulation properties. Preliminary data on mechanotransduction crosstalk between ECM stiffness and LSEC capillarization have been shown in this paper; we still need to carry out further studies, which would include establishing tunable hydrogels and 3D co-culture models with other cells in vitro. What is more, a translational study using a large-animal model with long-term follow up will be needed to assess efficacy (such as biomechano-pharmacological effects on anti-fibrosis) and safety before clinical translation.

At the same, due to its insolubility in water or general organic solvents, poor oral absorption and low bioavailability, silybin’s clinical efficacy is limited. Therefore, suitable silybin dosage forms should be designed to improve the targeting effect and more attention should be paid to the anti-ECM therapeutic effects of silybin, which are of great significance with respect to improving clinical applications. With the development of drug-carrier materials such as liposomes and polymers, silybin nanoparticles targeting the liver extracellular matrix can be constructed to improve its bioavailability against liver fibrosis. For instance, construct the silybin phospholipid complex can be considered to improve poor lipid solubility, or fat deposition can be inhibited by complexing phosphatidylcholine to promote the transport of silybin to the liver and improve the bioavailability of silybin in vivo.

In addition, the development of silybin dosage forms will require collaboration between researchers, clinicians, and industry partners to overcome technical and logistical challenges. This includes the development of cost-effective manufacturing processes for these dosage form, as well as the establishment of large-scale clinical trials to evaluate their efficacy in diverse patient populations.

## 4. Materials and Methods

### 4.1. Polyacrylamide Hydrogel Substrates

Polyacrylamide hydrogels (PAM) were prepared by mixing 40% acrylamide and 2% bis-acrylamide in different proportions and adding 10% ammonium persulfate and 1.25 μL tetramethylethylenediamine to initiate polymerization. The hydrogel stiffnesses for the experiments were set to 0.7, 4, and 10 kPa, which simulates the stiffnesses of the healthy, fibrotic, and drug-treatment liver tissues as measured via atomic force microscopy (AFM). Glass coverslips (Young’s modulus of ~1 GPa) were used as a control. The hydrogels were characterized using an elastic tensile tester, with the tensile length and speed set as 20% and 1%/min of the original length.

### 4.2. Extraction of Primary LSECs

C57BL/6 male mice aged 4 weeks were sacrificed to extract primary LSECs. The mice were anesthetized with 1% pentobarbital (1 mL/100 g of body weight (bw)) after fasting for 12 h. First, perfusion and digestion of the liver were performed in situ. The pre-perfusion solution (D-Hank’s buffer + Ethylene glycol tetraacetic acid + heparin sodium) and post-perfusion solution (Dulbecco’s modified Eagle’s medium (DMEM) + type IV collagenase + CaCl^2 +^ DNase I) were prepared in advance and pre-warmed at 37 °C. After perfusion, the liver was harvested, and the gallbladder was removed. Then the liver was gently shredded by gentle and repeated pipetting until the liver cells were completely and evenly dispersed and only connective tissue remained. The cell suspension was collected and centrifuged. The cell pellets were resuspended in DMEM and purified via Optiprep density gradient centrifugation (24%, 17.6%, and 11.2%). Finally, we used the adherent culture method to remove Kupffer and other contaminant cells, and obtained primary LSECs for our experiments.

### 4.3. Ca^2+^ Carrier A23187 to Induce NO Release

The primary LSECs were cultured on the different stiffness substrates for 24 h. Then, the medium was removed, and the cells were loaded with the DAF-FM DA fluorescent probe (final concentration: 5 μmol/L) in situ. After labeling, the cells were incubated in a 37 °C cell incubator for 20 min and then washed thrice with PBS to remove any unloaded probes. NO release was recorded using a confocal laser scanning microscope. Regions of interest were selected for real-time monitoring using live view. The fluorescence time series was imaged at 10 s/frame for 480 min. The Ca^2+^ carrier A23187 (final Concentration 1 μM) was added at 120 s. Fluorescence pictures were collected in real time and saved for statistical analysis.

### 4.4. Animal Preparation

C57BL/6J mice, male, 4–6 weeks old were purchased from Beijing Weitong Lihua Laboratory Animal Technology Co., Ltd., (Beijing, China). The breeding and experimental conditions of the experimental animals conformed to the “Administrative Regulations on Laboratory Animals” promulgated by the National Science and Technology Commission, and the protocol was approved by the ethics committee of the National Center for Nanoscience and Technology. The mice were housed in cages with no more than 5 mice per cage and had unrestricted access to autoclaved water and food. All mice were bred and kept under specific pathogen-free conditions.

### 4.5. Animal Models and Drug Intervention

The experiment consisted of control (healthy), model (fibrosis), and treatment (silybin treatment) groups, each with 6 mice. After adaptive feeding, model and silybin group mice were injected intraperitoneally with 5 µL/g bw of 10% (vol/vol) CCl_4_ (MacLean, C805332, Hangzhou, China) twice a week for 8 consecutive weeks to chemically induce liver fibrosis. The mice in the control group were injected with olive oil of equal volume. Silybin intervention began from the third week of injections, and the gavage dose of silybin for each mouse was 31.5 mg/kg/d (calculated from the human dosage for an average body weight of 60 kg, and the conversion factor between mice and humans being 9). The control and model groups were given the same volume of saline by gavage. The administration frequency was once a day for 8 weeks.

### 4.6. Evaluation of Liver Elasticity via Ultrasound

The mice were fasted with water for 6 h before the experiment and anesthetized via inhalation of 1–2% isoflurane. They were then placed on the monitoring platform in a supine position. The feet were fixed gently, and the hair on the front chest and whole abdomen was removed with depilatory cream to fully expose the liver area, while simultaneously applying a small amount of conductive glue on the exposed skin. A small animal ultrasound machine (Vevo, VisualSonics Vevo LAB, Tokyo, Japan) was used to detect the liver elasticity of living livers in mice. Care should be taken to ensure that the mice are fixed on the platform and connected to the physiological signal acquisition unit before scanning using B-Mode imaging.

### 4.7. Measurement of Young’s Modulus of Liver Tissue

Freshly isolated liver tissue was cut into pieces of about 5 mm × 5 mm × 3 mm, avoiding extrusion, embedded in OCT embedding medium, and flash frozen in liquid nitrogen. The pieces were cryosectioned to a thickness of 40 μm, carefully attached to glass slides, and then immersed in high-glucose DMEM supplemented with protease inhibitors. The Young’s modulus of the liver tissue was determined via bio-AFM (Nikon 5500, Tokyo, Japan) within 6 h. A graded transparent plate was pasted under the petri dish containing the sample with biocompatible glue to better locate the indentation points and AFM probe location. We examined at least 20 locations per sample, and each site was indented at 30 points within an area of 30 μm × 30 μm.

### 4.8. Tensile Testing of Liver Tissue

The freshly isolated left lobe of the liver was cut into blocks 2 mm × 4 mm × 10 mm. They were then fixed with clamps, and the stretch curves were recorded at a strain rate of 1%/s. Young’s modulus was determined by dividing the stress σ by the strain ε, at 20% strain.

### 4.9. Pathological Staining

Freshly isolated liver tissues were fixed in 4% (vol/vol) paraformaldehyde for more than 24 h, embedded in paraffin, and histopathologically sectioned via conventional methods. The histopathological sections underwent hematoxylin and eosin (HE), Sirius red, and immunofluorescence staining. The pathological changes were observed and photographed for analysis.

### 4.10. Environmental Scanning Electron Microscope (ESEM) Specimen Preparation

Perfusion was performed in situ, and the whole liver was extracted. The livers were cut into 5 mm × 5 mm × 3 mm pieces and soaked in 2.5% (vol/vol) glutaraldehyde for 24 h. The fixed tissues were washed thrice with phosphate buffered saline (PBS), followed by ethanol gradient dehydration (30–50–75–85–95–100% vol/vol, each gradient dehydration 15 min × 3 times). Then the samples were immersed in hexamethyldisilazane for 3 min, and placed in a vacuum desiccator for 25 min for evaporation. Before SEM, the liver tissues were vacuum-sprayed with gold film for 3–5 s. The preparation of cell samples for SEM was performed similarly to liver tissue samples. Briefly, the isolated LSECs were cultured on hydrogels for 24 h, the culture medium was aspirated, cells were washed thrice with PBS, and then they were fixed with 2.5% glutaraldehyde and stored overnight at 4 °C protected from light. The dehydration, evaporation, and other processes were the same as liver tissue sample preparation.

### 4.11. Western Blotting

The whole protein was extracted for western blotting. Phenylmethylsulfonyl fluoride (PMSF; Sigma, Saint Louis, MO, USA) stock solution was added to a pre-chilled radioimmunoprecipitation assay buffer (RIPA; Sigma, Saint Louis, MO, USA) to a final concentration of 1 mM and added to samples before protein extraction. The samples were lysed on ice for 20 min, and the collected lysates were centrifuged at 15,000 rpm for 15 min at 4 °C. The supernatants were collected, and the concentrations of proteins were analyzed. The bicinchoninic acid assay was performed to measure total protein concentration. Loading buffer (4×) was added to the samples to dilute the total protein concentration to 2 mg/mL, and boiled for 5 min to denature the proteins. Electrophoresis was performed after loading each well with 15 µg of total protein and then transferred onto a transfer membrane. Nonspecific blocking was performed with 5% nonfat milk powder for 1 h at room temperature. The membrane was incubated overnight at 4 °C with the primary antibody diluted in 5% bovine serum albumin in tris-buffered saline with tween-20 (BSA-TBST), followed by shaking gently for 40 min at room temperature with the secondary antibody, goat anti-rabbit IgG (H + L) HRP (Jackson, PA, USA), diluted in 5% BSA-TBST. The membrane was then washed thrice in TBST for 10 min each time. Enhanced chemiluminescence solution (ECL; Pierce, IL, USA) was used for protein visualization (commercial antibodies: eNOS Abcam Ab76198; VEGFA Abcam Ab46154; Caveolin-1 Santa cruz biotechnoloc Sc-53564; CD31 Abca Ab222783; GAPDH Beyotime Biotechnology AF5009; β-actin Beyotime Biotechnology AF0003).

### 4.12. Statistical Analysis

Data are presented as mean ± SEM. GraphPad Prism (version 8) software was used for statistical analysis. Data were analyzed using *t*-test and ANOVA with Bonferroni’s post-hoc test, and *p* < 0.05 was considered statistically significant.

### 4.13. Data Availability

The main data supporting the results in this study are available within the paper. Source data are provided with this paper. The raw and analyzed datasets generated during the study are too large to be publicly shared. More data are also available for research purposes from the corresponding authors on reasonable request.

## 5. Conclusions

Our study reveals ECM-induced mechanotransduction of capillarized LSECs through an NO-dependent pathway via a previously unrevealed mechanotransduction mechanism. The elucidation of this mechanism may offer precise biomechanics-specific intervention strategies targeting liver fibrosis progression.

## Data Availability

The data presented in this study are available on request from the corresponding author.
